# Sickle Cell Intrahepatic Cholestasis: Extremely Rare but Fatal Complication of Sickle Cell Disease

**DOI:** 10.7759/cureus.22050

**Published:** 2022-02-09

**Authors:** Arshan Khan, Bola Nashed, Mohamed Issa, Muhammad Zatmar Khan

**Affiliations:** 1 Internal Medicine, Ascension St. John Hospital, Detroit, USA; 2 Internal Medicine, Ascension St. John hospital, Detroit, USA; 3 Gastroenterology and Hepatology, Rutgers New Jersey Medical School, New Jersey, USA

**Keywords:** exchange transfusion in sickle cell intrahepatic cholestasis, sickle cell intrahepatic cholestasis prognosis, sickle cell intrahepatic cholestasis management, hepatic complication of sickle cell disease, sickle cell intrahepatic cholestasis

## Abstract

Sickle cell intrahepatic cholestasis (SCIC) is a rare but potentially fatal complication of sickle cell disease (SCD), with high mortality, observed mainly in patients with homozygous sickle cell anemia. Herein, we have reported a case of severe SCIC with a poor outcome despite aggressive measures including exchange transfusion and use of vasopressors.

The patient was admitted with generalized weakness, confusion, rigors, chills, and signs of hepatic failure, such as hyperbilirubinemia, hypoalbuminemia, and coagulopathy. There was no evidence of viral hepatitis or biliary obstruction. The patient received two exchange transfusions, but he continued to deteriorate clinically despite exchange transfusion and developed hemorrhagic shock and multiorgan failure. The patient was made comfort care as per family wishes. This case emphasizes the importance of early diagnosis of sickle cell intrahepatic cholestasis and poor prognosis despite aggressive measures.

## Introduction

Sickle cell disease (SCD) can affect multiple organ systems in the body. The hepatobiliary system is the most commonly affected within the digestive tract [1]. Liver manifestations include acute liver disorder (acute sickle hepatic crisis, hepatic sequestration crisis, vaso-occlusion, and liver infarction) and chronic liver disease due to hemosiderosis and hepatitis and possibly secondary to chronic silent microvascular occlusions [2,3]. Sickle cell intrahepatic cholestasis (SCIC) is an uncommon but potentially fatal hepatic manifestation of sickle cell disease [4,5]. This syndrome is characterized by sudden onset of right upper quadrant (RUQ) pain, hepatomegaly, mild elevation of transaminases, coagulopathy, and extreme hyperbilirubinemia [6]. Renal failure, coagulopathy, and bleeding diathesis are usually seen in severe cases [7].

The diagnosis requires exclusion of infectious etiologies of hepatitis and cholecystitis/cholangitis/choledocholithiasis [8]. Treatment ranges from supportive care to exchange transfusion and liver transplantation. The only effective management for severe sickle cell intrahepatic cholestasis is exchange transfusion to reverse the liver function [9]. Despite exchange transfusions, it can be fatal, and the transplant is the last resort. Herein, we have reported a case of severe SCIC with a poor outcome despite aggressive measures including exchange transfusion and use of vasopressors. 

## Case presentation

A 57-year-old male with a past medical history of sickle cell disease homozygous for hemoglobin (Hb) S without associated thalassemia/Hb C, not on any treatment, one to two episodes of sickle cell crisis in the last few years, and coronary artery disease was admitted to the hospital due to generalized weakness, confusion, rigors, and chills. His symptoms have been going on for about a week before the presentation. The patient was hypotensive, with a blood pressure of 95/57, and tachycardic, with a heart rate of 108 beats/minute; his respiratory rate was 39 breaths/minute, and his temperature was 97.6°F. On physical examination, the patient was confused and only oriented to self, the abdomen was distended, and generalized abdominal tenderness more in the right upper quadrant was present; the rest of the physical examination was unremarkable.

**Figure 1:**
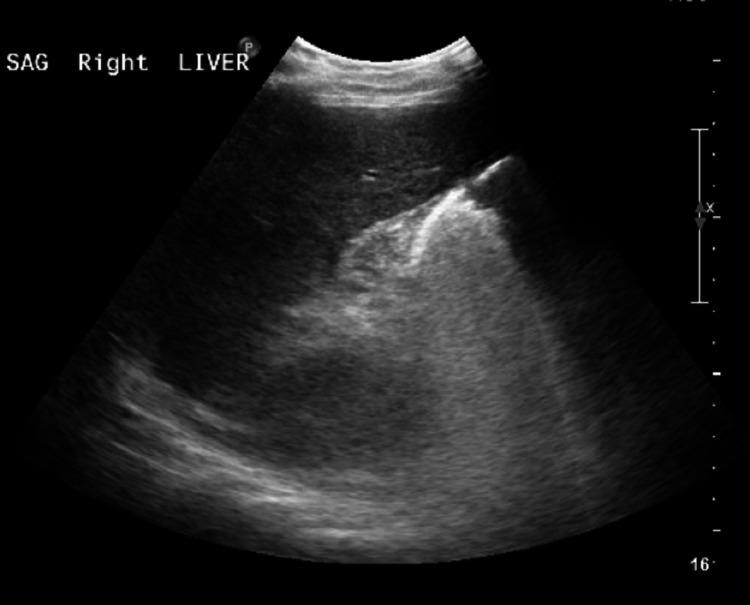
Ultrasound of the liver revealing findings compatible with cirrhosis, steatosis, and patent hepatic vasculature

The laboratory results on admission were as follows: creatinine, 2 mg/dL; sodium, 144 mEq/L; potassium, 3.2 mEq/L; albumin, 2.1 g/dL; WBC, 16,200 cells; Hb, 9.2 g/dL; platelets, 33,000/uL; international normalized ratio (INR), 3.3; activated partial thromboplastin time (APTT), 200 seconds; and GGT, 69/L. D-Dimer was elevated at 1,855, aspartate aminotransferase (AST) was 303 U/L, alanine aminotransferase (ALT) was 129 U/L, alkaline phosphatase (ALP) was 187 U/L, and total bilirubin was 49.7 mg/dL. Serology tests for hepatitis B and C and HIV were negative. The patient was started on vancomycin and Zosyn because of concern for ascending cholangitis (given leukocytosis, hyperbilirubinemia, and altered mental status). The patient was admitted to the ICU. ID and hepatology were consulted. Abdominal ultrasound was obtained, which revealed findings compatible with cirrhosis, steatosis, medical renal disease, and patent hepatic vasculature (Figure 1).

The patient received cryoprecipitate, fresh frozen plasma (FFP), and intravenous (IV) vitamin K for the correction of coagulopathy. Hematology was consulted for coagulopathy, and they recommended obtaining hemoglobin electrophoresis. The results showed a 35% (normal: 0%) hemoglobin S. Given the findings, he received two exchange transfusions to reduce the Hb S level by less than 30%. Meanwhile, he continued to deteriorate and developed hypoxic respiratory failure because of ARDS, hemorrhagic shock due to continued bleeding, and multiorgan failure. He was intubated for ventilatory support and started on vasopressors for hemodynamic support. His follow-up laboratory results showed an elevation in creatinine to 3.3 mg/dL, AST to 913 U/L, and ALT to 548 U/L; he remained coagulopathic with an INR of 3 and APTT of 158.7 seconds. He continued to have hyperbilirubinemia; the whole presentation was consistent with severe sickle cell intrahepatic cholestasis (SCIC). He was evaluated for liver transplant given the acute liver failure, but the hemodynamic status did not permit it to proceed further. Despite exchange transfusions and aggressive management with continued ventilatory support on high parameters, his vasopressor requirements increased. A palliative care consultation was obtained as per the family’s wishes. He was terminally extubated and passed away in peace.

## Discussion

Sickle cell hepatopathy is a term describing the involvement of the hepatobiliary system in sickle cell disease. Involvement can happen due to the vaso-occlusive phenomenon that signifies sickle cell disease or as a complication of frequent blood transfusions including viral hepatitis and gallstone formation. Acute hepatobiliary involvement due to vaso-occlusive crisis ranges in severity and characteristics, with sickle cell crisis and acute hepatic sequestration being the milder forms and acute intrahepatic cholestasis being the more severe form, which is our subject of focus here [10].

Vaso-occlusive sickling in hepatic sinusoids leads to ischemia, which leads to hepatocytic ballooning and biliary stasis, and biliary plugging in biliary radicles. Sinusoidal dilatation and erythrophagocytosis by Kupffer cells were also seen on liver biopsies [11]. It is to be noted that liver biopsies in patients with acute sickle cell crises were associated with a high risk of bleeding and death and do not change the management [12]. The clinical presentation of acute intrahepatic cholestasis differs from other forms of sickle cell hepatopathy in that it is more severe and accompanied by hepatic encephalopathy, coagulopathy, and renal failure [10].

Patients usually present with right upper abdominal pain, fever, leukocytosis, hyperbilirubinemia (mainly conjugated bilirubin > 15 mg/dL), marked elevation of liver enzymes up to >1000 IU/L that reflects ischemia, coagulopathy, and renal failure that developed probably secondary to sickle cell nephropathy or in response to multiorgan failure with hypotension as in our patient [13].

The treatment of sickle cell intrahepatic cholestasis is quite challenging, with mainly supportive treatment. Management is directed to not only treat the worsening liver function but also manage the end-organ damage accompanied by it. Patients with sickle cell intrahepatic cholestasis show evidence of associated coagulopathy and worsening kidney function [14-16].

Acute intrahepatic cholestasis is a fatal condition with supportive treatment as the mainstay of management [16]. Exchange transfusion is the main and first-line treatment, with a target to decrease the sickle cell burden, decrease the hemoglobin S fraction, and correct the underlying anemia [17,18]. They also reduce viscosity and interrupt the cycle of vaso-occlusion, stasis, and venous thrombosis in the microcirculation [18]. The target Hb S level is below 20%, with exchange transfusions for successful treatment of these episodes [14,17,18].

Patients showing evidence of coagulopathy are treated with fresh frozen plasma [17]. Temporary renal replacement therapy may be required for acute kidney injury, which may or may not improve with the improvement of liver function [14,15]. Our patient received exchange transfusions and FFPs. Due to his worsening hemodynamic status and uncontrolled bleeding, his prognosis was unfavorable, and he was made comfort care.

Alkhayyat et al. describe liver transplantation as a treatment option if supportive measures fail in two case series describing liver transplantation for patients suffering from liver dysfunction from sickle cell intrahepatic cholestasis [17]. The cases are from the pediatric population, with the first series describing six patients with one-, five-, and 10-year survival rates of 83.3%, 44.4%, and 44.4%, respectively. The second series described three pediatric patients with a reported survival rate of 66% [17]. Liver transplant is usually considered as a last resort, and there is significant periprocedural mortality and morbidity in these cases. Liver transplantation usually does not reverse the disease, and mortality continues to be high. Patients undergoing surgery may suffer from postoperative complications including graft failure and re-transplantation, sepsis, and immediate postoperative death. Those patients have been receiving monthly exchange transfusions after receiving liver transplantation [15,17]. Patients with sickle cell intrahepatic cholestasis are poor candidates for transplant due to multiorgan failure, as was the case with our patient.

Hydroxyurea increases Hb F levels and is often used to prevent complications in patients with sickle cell and decrease the need for blood transfusions, but its role in preventing SCIC is still uncertain, and it has not been shown to influence the frequency of hepatic sequestration crisis [15,17].

Irizarry et al. suggest that early cholecystectomy as an elective procedure simplifies the management of patients with sickle cell intrahepatic cholestasis by reducing the differential diagnosis in patients with sickle cell presenting with jaundice and RUQ pain [6].

## Conclusions

Sickle cell intrahepatic cholestasis is a rare but life-threatening manifestation of sickle cell disease. It should be one of the differentials in any patient with sickle cell who presents with abdominal pain, hyperbilirubinemia, and/or elevated liver enzymes. Despite exchange transfusion, patients have a poor prognosis, and physicians should have a high index of suspicion and refer for early transplant if the exchange transfusion does not work.

## References

[1] Shah R, Taborda C, Chawla S (2017). Acute and chronic hepatobiliary manifestations of sickle cell disease: a review. World J Gastrointest Pathophysiol.

[2] Ebert EC, Nagar M, Hagspiel KD (2010). Gastrointestinal and hepatic complications of sickle cell disease. Clin Gastroenterol Hepatol.

[3] Banerjee S, Owen C, Chopra S (2001). Sickle cell hepatopathy. Hepatology.

[4] Guimarães JA, Silva LC (2017). Sickle cell intrahepatic cholestasis unresponsive to exchange blood transfusion: a case report. Rev Bras Hematol Hemoter.

[5] Collen BR, Albert D, Smith C (2016). Acute sickle cell intrahepatic cholestasis (SCIC) successfully treated with exchange blood transfusion. Am J Gastroenterol.

[6] Irizarry K, Rossbach HC, Ignacio JR (2006). Sickle cell intrahepatic cholestasis with cholelithiasis. Pediatr Hematol Oncol.

[7] Costa DB, Miksad RA, Buff MS, Wang Y, Dezube BJ (2006). Case of fatal sickle cell intrahepatic cholestasis despite use of exchange transfusion in an African-American patient. J Natl Med Assoc.

[8] Brunetta DM, Silva-Pinto AC, do Carmo Favarin de Macedo M (2011). Intrahepatic cholestasis in sickle cell disease: a case report. Anemia.

[9] Hosiriluck N, Rassameehiran S, Argueta E, Tijani L (2014). Reversal of liver function without exchange transfusion in sickle cell intrahepatic cholestasis. Proc (Bayl Univ Med Cent).

[10] Theocharidou E, Suddle AR (2019). The liver in sickle cell disease. Clin Liver Dis.

[11] Ahn H, Li CS, Wang W (2005). Sickle cell hepatopathy: clinical presentation, treatment, and outcome in pediatric and adult patients. Pediatr Blood Cancer.

[12] Zakaria N, Knisely A, Portmann B, Mieli-Vergani G, Wendon J, Arya R, Devlin J (2003). Acute sickle cell hepatopathy represents a potential contraindication for percutaneous liver biopsy. Blood.

[13] Chitturi S, George J, Ranjitkumar S, Kench J, Benson W (2002). Exchange transfusion for severe intrahepatic cholestasis associated with sickle cell disease?. J Clin Gastroenterol.

[14] Khan MA, Kerner JA (2011). Reversal of hepatic and renal failure from sickle cell intrahepatic cholestasis. Dig Dis Sci.

[15] Malik A, Merchant C, Rao M, Fiore RP (2015). Rare but lethal hepatopathy-sickle cell intrahepatic cholestasis and management strategies. Am J Case Rep.

[16] Kwun Lui S, Krasinskas A, Shah R, Tracht JM (2019). Orthotropic liver transplantation for acute intrahepatic cholestasis in sickle cell disease: clinical and histopathologic features of a rare case. Int J Surg Pathol.

[17] Alkhayyat M, Saleh MA, Zmaili M (2020). Successful liver transplantation for acute sickle cell intrahepatic cholestasis: a case report and review of the literature. World J Hepatol.

[18] Delis SG, Touloumis Z, Bourli A, Madariaga J, Dervenis C (2006). Can exchange transfusions treat postoperative intrahepatic colestasis in patients with sickle cell anemia?. Transplant Proc.

